# Do rats (*Rattus norvegicus*) perceive octave equivalence, a critical human cross-cultural aspect of pitch perception?

**DOI:** 10.1098/rsos.221181

**Published:** 2024-05-29

**Authors:** Bernhard Wagner, Juan Manuel Toro, Ferran Mayayo, Marisa Hoeschele

**Affiliations:** ^1^ Austrian Academy of Sciences, Acoustics Research Institute, Vienna 1040, Austria; ^2^ Passeig de Lluís Companys, Institució Catalana de Recerca i Estudis Avançats (ICREA), Barcelona 08010, Spain; ^3^ C. Ramon Trias Fargas, Universitat Pompeu Fabra, Barcelona 08005, Spain

**Keywords:** octave equivalence, musicality, rats, auditory perception, music

## Abstract

Octave equivalence describes the perception that two notes separated by a doubling in frequency have a similar quality. In humans, octave equivalence is important to both music and language learning and is found cross-culturally. Cross-species studies comparing human and non-human animals can help illuminate the necessary pre-conditions to developing octave equivalence. Here, we tested whether rats (*Rattus norvegicus*) perceive octave equivalence using a standardized cross-species paradigm. This allowed us to disentangle concurring hypotheses regarding the evolutionary roots of this phenomenon. One hypothesis is that octave equivalence is directly connected to vocal learning, but this hypothesis is only partially supported by data. According to another hypothesis, the harmonic structure of mammalian vocalizations may be more important. If rats perceive octave equivalence, this would support the importance of vocal harmonic structure. If rats do not perceive octave equivalence, this would suggest that octave equivalence evolved independently in several mammalian clades due to a more complex interplay of different factors such as—but not exclusively—the ability to vocally learn. Evidence from our study suggests that rats do perceive octave equivalence, thereby suggesting that the harmonic vocal structure found in mammals may be a key pre-requisite for octave equivalence. Stage 1 approved protocol: the study reported here was originally accepted as a Registered Report and the study design was approved in Stage 1. We hereby confirm that the completed experiment(s) have been executed and analysed in the manner originally approved with any unforeseen changes in those approved methods and analyses clearly noted. The approved Stage 1 protocol can be found at: https://osf.io/gvf7c/?view_only=76dc1840f31c4f9ab59eb93cbadb98b7.

## 1. Introduction

Octave equivalence is a phenomenon in human hearing that describes the perception that sounds are separated by a doubling in frequency (or, in Western music theory terminology, an ‘octave’) sound similar. The shared quality that makes such notes sound similar is also referred to as ‘pitch chroma’. Importantly, notes can also sound more similar the closer they are in frequency, a phenomenon known as pitch height. Pitch height and pitch chroma both work to affect which notes we perceive as similar, and notes that share the same pitch chroma, that are separated by an octave, can in some cases be rated as more similar than notes that are close in terms of pitch height [1–3]. As such, our perception of octave equivalence is the reason notes with different absolute pitches (e.g. 220, 440 and 880 Hz) are, in Western music theory, all referred to by the same name (e.g. ‘A’) only adding the respective octave in cases where the precise frequency is needed (e.g. ‘A3’, ‘A4’, ‘A5’). Octave equivalence is, however, not only present in Western music; the octave has been recognized as a cross-culturally common basis of pitch perception ([4,5]; but see [6]). In practice, octave equivalence is what allows people with different vocal ranges to sing the same melody in unison, achieving maximum harmonic overlap and perceptual merging of multiple voices.

Besides music, octave equivalence is also important to human speech. It has been shown that young children imitating words from a voice with a fundamental frequency outside their vocal range (i.e. too low for the child to reproduce) will spontaneously transpose the sound by an octave [7–9]. This is not an arbitrary transposition: in the case where reproduction of the fundamental frequency is not possible, reproducing a harmonic sound such as the human voice by transposing it by an octave will result in a sound that shares as many overtones with the original sound as is possible [10]. As such, the resulting sound is harmonically most similar to the original sound. Critically, this association, while emerging from the harmonic series, appears to be so strong that humans perceive octave equivalence even with tones that do not contain harmonics (i.e. sine waves [2]).

The spontaneous use of octave equivalence in very young children further suggests that octave equivalence is unlikely to purely be a cultural phenomenon. There is some debate about whether this is cross-culturally true in humans because Jacoby *et al*. [6] found no evidence of the native Amazonian Tsimane’ producing octaves in a singing task, where Western participants did. However, in a subsequent experiment, the Tsimane’ too showed perceptual merging of notes with small integer frequency ratios [11]. This suggests that at least sensitivity to shared harmonic information, which is especially apparent in the octave, is present cross-culturally.

In sum, octave equivalence may be an important part of the unique sets of skills that enable human speech as well as music. Forming a better understanding of it could be key to comprehending how human language and music evolved. One way to understand the evolutionary origins and function of octave equivalence is through comparative research with non-human animal species. Comparative studies with model species allow for control of potential effects of enculturation in a way that is not possible in studies with human participants [12]. But perhaps even more importantly, cross-species comparative studies can be used to assess whether octave equivalence perception is a common trait among non-human animals and therefore has ancient evolutionary origins or whether it is limited to a specific clade or unrelated groups that share a particular ecological niche. If octave equivalence is rare but numerous species from differing clades all use octave equivalence in similar contexts, we may be able to deduce a common evolutionary function of octave equivalence across species.

An initial hypothesis about the evolutionary function of octave equivalence was that humans developed it to aid in vocal learning. Vocal learning is the ability to learn new vocalizations from conspecifics (or, in some cases, other sounds in their environment) and includes the human ability to learn language. Certainly, this hypothesis would appear logical as octave equivalence is used in human vocal learning as reviewed above. However, the comparative research regarding octave equivalence perception in non-human animals has so far not supported this hypothesis. Only two species have so far been at least tentatively shown to perceive octave equivalence, the first being rhesus macaques, *Macacca mulatta* (using a musical melody paradigm [13]) and the other being bottle-nosed dolphins, *Tursiops truncatus* (documented by spontaneous vocal imitation [14]). While dolphins are vocal learners, rhesus macaques (and indeed, other primates except humans) are not, making vocal learning an unlikely candidate for the origins of octave equivalence. Additional research has also found no evidence of octave equivalence in vocal learning avian species including black capped chickadees (*Poecile atricapillus* [15]) and budgerigars (*Melopsittacus undulatus* [16]). Cynx [17] also found no evidence for octave equivalence in vocal learning European starlings (*Sturnus vulgaris*) but the paradigm used was later shown to also not demonstrate octave equivalence with human participants [2]. As such, it appears that the data obtained in non-human animals so far does not support the idea that octave equivalence is generally linked to vocal learning.

Instead, the existing data better supports several other hypotheses. The first is that octave equivalence *perception* may be a trait common across mammalian orders with octave equivalence *production* in vocal learning mammalian species possibly stemming from this more common ability.

Mammals may benefit more generally from octave equivalence for a number of reasons. One reason is that mammalian species tend to have more pronounced harmonic information in their vocalizations compared to the birds that have been studied in this context [18]. Clear harmonic information is important for octave equivalence, because octave equivalence is essentially a successful generalization to a signal with similar harmonic information. If harmonic information is not prominent, then octave equivalence would be a poor tactic to recognize a signal. Another reason to suspect mammals more generally may perceive octave equivalence is that they tend to have longer juvenile developmental phases, especially in terms of body size (and thereby, also vocal range). If members of a species need to generalize across vocalizations of members with differing vocal ranges, harmonic information could help them do so. The importance of these traits could explain why octave equivalence may be common in mammals and not in other classes such as, e.g. birds [18].

However, given that the only empirical evidence of octave equivalence to date is in rhesus macaques [13] and dolphins [14], a second hypothesis is that octave equivalence evolved independently in primates and dolphins. Thus, more research is needed in other (especially mammalian) species. Ideally, species that allow for using larger sample sizes should be studied because earlier work relied on one [14] or two [13] individuals.

Rats (*Rattus norvegicus*) are perhaps the most common laboratory animal which makes testing larger sample sizes easier. Testing rats for perception of octave equivalence could help disentangle some of the opposing current hypotheses. Because rats are not a vocal learning species as far as existing studies have found, if they do perceive octave equivalence. it would constitute further support against the vocal learning hypothesis. It also would support our first alternative hypothesis that octave equivalence is common among mammals. Indeed, rat vocalizations feature clear harmonics [19] and juvenile vocalizations are higher frequency than those of adults [20]. If rats do not show octave equivalence, this would support our second alternative hypothesis that octave equivalence is a case of analogous evolution in dolphins and primates that is not generally found in mammals.

As rats are such a common model species, it may be unsurprising that there is existing research on octave equivalence in this species. But, as previously pointed out by Burns [4], a study on octave equivalence in rats by Blackwell & Schlossberg [21] may have had harmonic distortion and thereby may have presented the octave during training. During testing, when the rats responded to the octave, it was unclear whether they were simply generalizing to features included in the training stimuli. Crucially, as stated above, human octave equivalence, while emerging from the harmonic series, is even perceived with pure tones that do not have harmonic content (i.e. sine waves). A conclusive test of octave equivalence in non-human animals should show the same generalization. In another study on rat auditory perception, D’Amato & Salmon [22] showed that rats are able to retain the discrimination of two melodies after one of the melodies was octave transposed. However, this could be for any number of reasons, only one of which is octave equivalence (others include responding based solely on the untransformed melody and use of relative pitch), and the authors also make no claim that the rats were using octave equivalence to solve the task. As such, from the existing literature, conclusions regarding rats’ perception of octave equivalence are not yet possible.

Beyond the problems detailed above, there is also another issue with these earlier studies, namely that neither allows for direct comparisons with humans. This can lead to faulty conclusions. For example, a paradigm by Cynx [17] concluded European starlings did not have octave equivalence but later, testing showed the paradigm could also not demonstrate octave equivalence in humans [2]. Therefore, in the study presented here, we endeavoured to test rats for octave equivalence using a verified paradigm that allows for direct comparison with human data. Hoeschele *et al*. [2] developed a standardized, non-verbal operant conditioning go/no-go task that reliably demonstrates octave equivalence in humans and which can readily be used to test non-human animals as well. Participants in this study received no instructions and learned how to solve the task using trial and error just as a non-human subject would. As such, the paradigm allows for reliable comparison between humans and non-human animals. Such comparative testing has since been carried out with black-capped chickadees [15] and budgerigars [16]—allowing for comparison with those species as well. This paradigm trains individuals to respond to a subset of notes from one octave but not to the rest of the notes in the same octave. In a subsequent test with unrewarded notes from a higher octave, human individuals responded significantly more to the notes corresponding to the lower octave notes that they had previously been trained to respond to. If rats perceive octave equivalence, they would be expected to show the same pattern of responses.

As such, our study will disentangle two opposing hypotheses. One is that octave equivalence is a common trait among mammals. The other is that octave equivalence is an adaptation that evolved independently in some species. If rats perceive octave equivalence, we expect the rats to respond more to notes an octave above the notes presented during familiarization as opposed to other novel notes. This would support the hypothesis that octave equivalence is common among mammals. Alternatively, if rats do not perceive octave equivalence, we expect not to find a difference in responding to the octave of familiarized and non-familiarized notes. This would support the hypothesis that octave equivalence is an adaptation that evolved separately in several species.

## 2. Methods

### 2.1. Subjects

We trained 40 female Long-Evans rats (*R. norvegicus*) of three months of age at the start of the study. As in previous successful studies (e.g. [23,24]), we only worked with female rats as they are easier to house and have higher response rates than males. All subjects were naïve to both the stimulus sets and the apparatus used here. From earlier studies, we expected that using this number of individuals would result in enough statistical power to allow for strong conclusions [23,24]. If a rat failed to interact with the apparatus entirely, it would have been replaced with another that was naïve to the stimuli and the apparatus. However, this did not occur. The rats are housed at the Parc de Recerca Biomedica de Barcelona where they have water ad libitum. Their body weight during the experiment was maintained at 90–95% of their free-feeding weight because the experiment involves food rewards and satiation would obstruct successful training. Sucrose pellets were used as a reward food in every session (45 mg purified pellets; F0021, BioServ). Sessions occurred daily, with each session lasting for no more than 30 min.

### 2.2. Apparatus

The general setup for this study is the same as in previous studies from the same laboratory (e.g. [23,24]). Rats were trained and tested in modular response boxes (reference LE1005; Panlab S. L., Barcelona, Spain), equipped with a pellet feeder. Attached to the feeder was a photoelectric detector that registers rats’ nose-poking responses. Every response box was acoustically isolated by being positioned within a larger soundproof box. Auditory stimuli were presented via Electro Voice (s-40) speakers which were located adjacent to the modular response boxes. Stimulus presentation was controlled via a custom-made program (RatboxCBC v.2) which also recorded nose-poking responses and automatically dispensed food reward.

### 2.3. Stimuli

We used sine wave tones constructed at a standard 16-bit 44.1 kHz sampling rate. These sine wave tones had a duration of 440 ms and were ramped upwards at the onset and downwards at the offset for 5 ms so as to avoid transients [2,15]. For training (see below), we used four notes of octave six, namely E6 at 1320.4 Hz, F6 at 1398.9 Hz, F#6 at 1482.1 Hz and G6 at 1570.23 Hz. For testing (see below), we additionally used 12 notes of octave seven beginning with C7 at 2096.00 Hz and ending with B7 at 3956.72 Hz. The frequency of all the notes used for the experiment was calculated using the following formula: *F*_x_ = 2^(1/12)^ × *F*_*x*−1_, where C4 = 262 Hz [15]. The stimuli were the same in every aspect as those used in previous experiments with humans [2], black-capped chickadees [15] and budgerigars [16], except that in previous studies, training was done in octave four and testing in octave five. We shifted the stimuli up by two octaves in this case to make stimuli as relevant as possible to rats’ auditory perception (which favours higher frequencies than humans), while keeping absolute frequencies as close as possible to the original study to still allow for comparability. Rats' greatest hearing sensitivity is around 40 000 Hz and a large proportion of their communication occur in ultrasound (e.g. [19,25,26]). It is perhaps worth noting here, that the frequencies of all described stimuli are well within rats’ hearing range at the used presentation amplitudes (e.g. [27,28]). In fact, we chose this range of stimuli as rats' audible (to humans) vocalizations occur around 2–4 kHz (which includes most of the chosen octave ranges) and these vocalizations in particular feature harmonic content [19]. As such, these stimuli are in the ideal range for our purposes. Additionally, stimuli were played at two different amplitudes intermittently: soft (categorized as ‘S’) at 70 dB SPL and loud (categorized as ‘L’) at 80 dB SPL, so that loudness could not be confounded with pitch as can happen in humans [29].

To be certain of the absence of perceivable harmonics during training and testing, stimuli were additionally recorded at the position of the individual in the test using a commercial audio recorder (Sony ICD-PX240). Subsequent spectral analysis of the recorded stimulus using ‘Praat’ (GPL) revealed that the speakers did generate some harmonic distortion, producing a first and second overtone for the sounds. The first overtone had an intensity of 17 dB and the overtone had an intensity of 13 dB. Both of these overtones were inaudible to rats according to the audiograms in Gourevitch & Hack [27] and Heffner *et al*. [28] and could therefore not influence the results.

### 2.4. Procedure

The procedure used in this study is based on Hoeschele *et al*. [2], using a well-established and replicated paradigm applicable across species to test for octave equivalence. The paradigm has been modified only slightly to cater to rats as test species. Specifically, we used a testing method where rats are not required to respond by using, e.g. a lever or a touchscreen. Past studies in our laboratory have been more successful with such an approach rather than a go/no-go approach (e.g. [24,30]).

#### 2.4.1. Familiarization

Each session consisted of placing a single rat in a response box. Prior to the actual experiment, rats learned that they could receive a food reward upon putting their nose into the feeder. This usually required between one and two sessions of training. Once they learned this behaviour, we used a familiarization procedure to habituate them to four notes for 20 sessions (running one session per day). Here, a reward could only be received by nose-poking after stimulus playback had completed. Subjects were presented with the middle four notes of octave six: E, F, F# and G. Each familiarization note was then presented 25 times per session. The order of presentation of the notes was random, with the only restriction being that the same note cannot be presented more than three times in a row. There was a 10 s interval of silence between individual notes. We used a 10 s interval because this is the interval that previous studies in the same laboratory used to successfully demonstrate acoustic stimulus discrimination [24,30]. The rats received a food reward of one pellet every time they placed their nose into the feeder during that interval. After 20 sessions were completed, rats were transferred to the testing phase.

#### 2.4.2. Testing

We ran two test sessions, A and B, with a day of familiarization training between tests (3 days total). The order of the sessions was counterbalanced across subjects. Both test sessions included 18 unrewarded probe trial stimuli (6 notes × 3 trials each) plus 20 rewarded familiar stimuli. Two of the three probe trials for three of the probe notes were presented at 70 dB SPL (S) and one of the trials at 80 dB SPL (L). For the other three probe notes, two of the probe trials were presented at 80 dB SPL (L) and one of the trials at 70 dB SPL (S). Which rats heard which loudnesses for which notes was pseudo-randomized such that all notes were heard at both loudnesses an equal number of times across all rats. In test A: C7, D7, E7, F#7, G#7 and A#7 were probe tones (every other note in ascending order of octave 7). In test B: C#7, D#7, F7, G7, A7 and B7 (the remaining tones one semitone up from the tones presented in test A) were probe tones. The rationale behind this is that if subjects do perceive octave equivalence they should respond the most to the notes E7, F7, F#7 and G7 in octave 7 as these are an octave above the notes presented during familiarization in octave 6. That is, when an unfamiliar stimulus is presented during testing, the attention of the rats may be attracted to the stimuli and the poking will be inhibited. As such, the critical measure during testing is actually a lack of nose-poke responses, putatively because they detected the change and are distracted by the sound. This is in fact what has been observed in previous studies (e.g. [23,24]), see also infant head turning experiments where the critical measure is also attention (e.g. [31–33]). Presentation of stimuli during testing was pseudo-randomized such that the same stimulus did not occur more than two times in a row. There were no more than three rewarded or unrewarded stimuli in a row and a regularly alternating pattern of stimuli was avoided.

Importantly, we were not concerned about a potential ceiling effect during the test. A scenario in which the animals produce many responses to transposed notes while not producing any response to semitones would provide very strong evidence of the presence of octave equivalence in rats. We also considered a floor effect—where animals learn nothing during familiarization—to be highly unlikely based on previous experience with the proposed paradigm. However, to control for this possibility, we included four unrewarded probe trials of training stimuli *during testing*. If an individual animal did not respond at all during these probe trials (i.e. 0 out of all training stimuli probe trials), their data were excluded from analysis. This occurred for one animal. Including training stimulus probe trials also allowed us to make sure that rats learned to respond to the familiarization stimuli. In previous studies, 20 trials of familiarization were enough to trigger a differential response during testing [23,24]. Using unrewarded training stimulus probe trials further allowed us to compare response to training notes with response to other notes for stronger conclusions with regards to our hypotheses. See table 1 for an interpretation of possible response outcomes: if rats show no discrimination, they should respond randomly to all stimuli. In that case, pitch information (and therefore also octave equivalence) would appear to be less salient to rats than it is to species such as humans. This outcome, however, appears highly unlikely due to the aforementioned previous studies where rats indeed learned similar (and more difficult) auditory discriminations [23,24].

**Table 1.** Expected relative response to stimulus types for rats showing no discrimination, rats showing discrimination and octave equivalence and rats showing discrimination but no octave equivalence.

	familiarized tones	octave test tones	non-octave test tones
no discrimination learned	=	=	=
discrimination but no octave equivalence perception	>	<	<
discrimination and octave equivalence perception	>>	>(>)	<

Rats that do not perceive octave equivalence will respond significantly more to training stimuli than to all other stimuli. Rats that do perceive octave equivalence will respond significantly more to training stimuli and their octaves than to other stimuli.

### 2.5. Power analysis

We conducted a power analysis before running our experiment, to make sure our design allowed for conclusive results.

In a previous highly similar study with humans ([34]; note, however, that this study used an operant paradigm) we found octave transposed ‘S−’ notes to be responded to at chance level, i.e. of 0.5. We found that octave transposed ‘S+’ notes were responded to at a probability of 0.65. The odds ratio of reaction to ‘S+’ notes was therefore 0.65/0.35 = 1.857. For a conservative estimate, we therefore conducted power analysis under the assumption that rats would show a less strong response probability than humans. Setting the ‘S+’ response probability to 0.6, we arrived at an odds ratio of reaction to ‘S+’ notes of 0.6/0.4 = 1.5. Different species differ in their raw number of responses (see, e.g. the comparison of go-biased budgerigars in Wagner *et al*. [16] with no-go-biased chickadees in Hoeschele *et al*. [15]). As such, we considered the odds ratio to be more important here than the raw number of responses.

Our results analysis made use of a logistic regression model to estimate the effect of ‘S+’ on reaction. In the testing phase, rats are presented with 12 ‘S+’ and 24 ‘S−’ notes. For unrewarded notes, we assumed that the probability of a reaction is 0.5 (as we do not know whether rats are go-biased or no-go-biased) and for rewarded notes a probability of 0.6, corresponding to the desired odds ratio of 1.5 and an effect of ‘S+’ on the log-odds ratio of 0.4.

We simulated the power for a test on an effect of ‘S+’ with *n* = 40 rats each of which has 36 (12 ‘S+’, 24 ‘S−’) trials under two scenarios: in the first, we assume homogeneous reaction probabilities across rats, whereas in the second, we allow for heterogeneity of reaction probabilities across rats. We simulated 10 000 datasets for each scenario, respectively.

In the first scenario at significance level *α* = 0.05, we obtained a (simulated) power of 0.95. In the second scenario, we allowed for heterogeneity across subjects. We assumed the same fixed effect of 0.4 and subject-specific random intercepts following a standard normal distribution on the log odds ratio. This means that the subject-specific reactions on ‘S−’ notes with a probability of 0.95 were in the range of 0.123–0.877, whereas for the rewarded notes, the corresponding probabilities were in the range of 0.174–0.914. In this scenario, a reaction to ‘S+’ notes of at least 0.6 (corresponding to the desired odds ratio of 1.5) is detected in a logistic regression model with subject-specific intercepts with a power of 0.911.

For the case of having to remove some individuals, we simulated the power for a test on an effect of ‘S+’ with *n* = 30 rats each of which has 36 trials. We repeated the power analysis for both scenarios and obtained a power of 0.8798 for the first scenario and of 0.8156 for the second scenario. As such, the numbers we chose for subjects and trials here were appropriate for showing the effect that we expected.

Note: see electronic supplementary material for the code used for this power analysis [35].

### 2.6. Analysis

For analysis, we compared the 76 trials from tests A and B across all individuals. 40 of these trials were octave six notes (20 trials per session) and 36 of these trials were octave 7 notes (3 trials each of 12 notes). The data were analysed using a logistic regression with the proportion of responses to each note as the target variable. We used a logistic regression because it is the standard analysis for binary target variables where we want probability to be modelled in terms of covariates [36]. We included the covariates, ‘test order’ (test A/test B) and ‘volume’ (loud/silent). Furthermore, we controlled for individual ID by including ‘subject identity’ as a nominal covariate. This analysis allowed us to compare responses to the different octave seven test notes and can thereby illuminate whether habituated notes (i.e. E, F, F#, G) were responded to significantly more than non-habituated notes in the test octave.

## 3. Results

**Figure 1:**
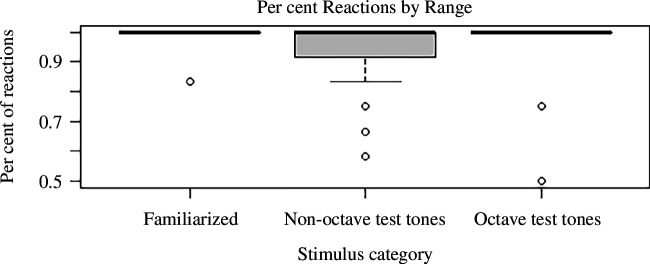
Per cent response by stimulus category for all tested individuals. Black bar represents median, circles represent outliers, i.e. values more than 1.5 times the interquartile range away from the box outliers and shown as circles. Whiskers extending from the box signify minimum and maximum of the remaining values.

Out of the 40 rats we trained, one was excluded for not producing any nose-pokes in training. For the remaining *n* = 39 rats, as described above, we performed a logistic regression with proportion of responses out of all presentations to each note as the target variable and covariates ‘test order’ (test A/test B), ‘volume’ (loud/silent) and ‘subject identity’ as a nominal covariate. Most crucial was covariate ‘stimulus category’ which compared per cent responses to three types of stimuli: (i) the intermittently presented unrewarded familiarization stimuli (‘familiarized’ including E6, F6, F#6 and G6), (ii) the octave seven counterparts to familiarization stimuli (‘octave test tones’ including E7, F7, F#7 and G7) and (iii) the remaining octave seven tones (‘non-octave test tones’). We found a significant difference in response to octave test tones and non-octave test tones (*p* = 0.002) with subjects responding less to non-octave test tones. We found no significant difference in response to octave test tones and familiarized tones (*p* = 0.26). There was no significant effect for the other covariates (all *p*’s > 0.05). See table 2 in the Appendix for detailed results including estimated coefficients, their standard errors and the *p* values from the standard Wald test. Figure 1 shows a comparison of per cent responses to the tones of the three different stimulus categories.

**Table 2.** Detailed results from logistic regression including estimated coefficients, their standard errors and the *p* values from the standard Wald test. All our models were fitted in R-software. For the covariates, we set the following categories as baseline values: stimulus category: octave test tones, loudness: 80 dB SPL (L), order: first test.

	estimate	std. error	*z* value	Wald test *p*
(intercept)	21.3849081339	2570.8673553339	0.0083181686	0.9933631382
non-octave test tones	−1.5563657636	0.4975600627	−3.1279957542	0.0017600272
familiarized	1.2534088469	1.1115377719	1.1276349563	0.2594741175
volume ‘S’	0.371673454	0.6914172871	0.3430162216	0.7315862566
test order: test 2	−0.3169561847	0.266945357	−1.1873448122	0.2350916389
non-oct. test tones: volume ‘S’	1.0359459989	0.7713392977	1.3430483861	0.1792563067
familiarized: volume ‘S’	−1.9307586315	1.3117276459	−1.4719203621	0.1410423843

### 3.1. Additional analysis

**Figure 2:**
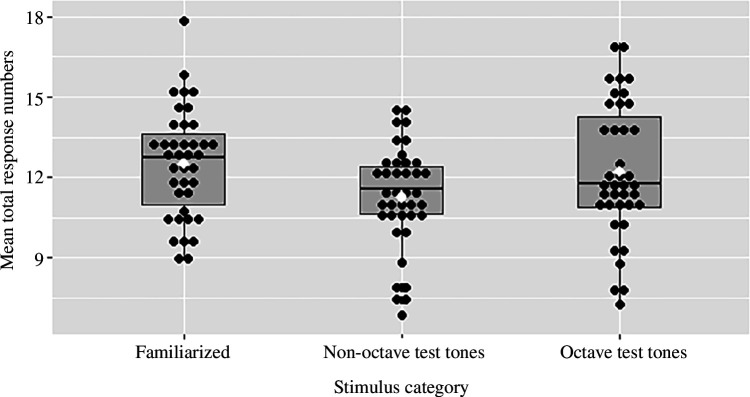
Mean of total number of responses to notes in each stimulus category for all individuals where individuals are black dots, white dot is overall mean per stimulus category and black bar is the median.

As the experimental programme recorded not only whether a stimulus was responded to but also how many nose-pokes occurred in the 10 s period after stimulus presentation, we ran an additional analysis using a Poisson regression with the same covariates as specified above. This provided a more continuous measure of responding to the stimuli. Here too, we found a significant difference in total response number to octave test tones versus non-octave test tones (*p* < 0.001) with subjects responding less to non-octave test tones. We found no significant difference in total response number to octave test tones and familiarized tones (*p* = 0.85). Additionally, here we found an effect for test order (*p* < 0.001) with fewer total responses in later tests. We also found a significant effect for the interaction of loudness and non-octave test tones with such tones getting higher total response numbers if they were softer (*p* < 0.001). There was no significant effect for the other covariates (all *p*’s > 0.05). See table 3 in the Appendix for detailed results including estimated coefficents, their standard errors and the *p* values from the standard Wald test. Figure 2 shows a comparison of total responses to the tones of the three different stimulus categories.

**Table 3.** Detailed results from Poisson regression including estimated coefficients, their standard errors and the *p* values from the standard Wald test. All our models were fitted in R software. For the covariates, we set the following categories as baseline values: stimulus category: octave test tones, loudness: 80 dB SPL (L), order: first test.

	estimate	std. error	*z* value	Wald test *p*
(intercept)	2.6172240038	0.0464180311	56.3837789865	0
non-octave test tones	−0.1800882801	0.0232712699	−7.7386528941	1.00475672176356e^−14^
familiarized	0.005573868	0.029052695	0.1918537328	0.84785678
volume ‘S’	−0.0170827523	0.0261408362	−0.6534891283	0.513440996
test order: test 2	−0.0476704513	0.0140668982	−3.388838858	0.0007018925
non-oct. test tones: volume ‘S’	0.1492950843	0.0326163295	4.5773110189	4.7099094792829e^−06^
familiarized : volume ‘S’	−0.0590095405	0.0416401884	−1.4171295283	0.1564450608

## 4. Discussion

Here, we conducted a study testing whether rats perceive octave equivalence. Rats were familiarized with tones from octave 6 and then presented with novel tones from octave 7. Animals that perceive octave equivalence should respond similarly to the familiarization stimuli and novel tones that are octaves of the familiarization stimuli, but measurably different to other novel tones. This is precisely what we found. Rats in our study showed no discrimination between habituated tones and their octave counterparts but responded significantly less to other novel tones as measured by percentage of binary responses, as well as total nose-pokes after stimulus presentation. This result therefore constitutes strong evidence of octave equivalence perception. The plausibility of this conclusion is strengthened by our study designs’ comparability to studies with human participants [2,34] on which our paradigm was based. It is also worth mentioning that our results are in line with an earlier study that implicitly suggested rats may perceive octave equivalence although this was not directly tested [37]. They further support the conclusions from Blackwell & Schlosberg’s [21] study of octave equivalence in rats where technical issues in the study design rendered their results undependable.

That we did not find the effect in each single individual does not detract from the validity of this result. This is evident from statistical analysis, but it is worth mentioning that even in the studies with humans that this work is based on, we did not find octave equivalence in every single participant [2,34]. There are multiple explanations for why individual rats may not have shown octave equivalence in testing, the most apparent explanation being a more pronounced ‘go-bias’, driving bolder animals to generally respond more, regardless of specific stimulus (see also [16]). Yet even if for some—at this point unaccountable—reason only a proportion of rats perceived octave equivalence, this would still not detract from our main finding that octave equivalence can occur in this species without external influence.

Note that none of the training implemented here was used to make the rats *perceive* octave equivalence. The entirety of our training is implemented to be able to demonstrate a perception that is independent of our training and therefore pre-existing. As rats in our laboratory are not exposed to human music at all, this suggests that the phenomenon of octave equivalence occurs naturally—i.e. without exposure to human musical culture or enforced training. This is in line with our underlying assumptions.

We conducted this study to test concurring hypotheses on the evolution of octave equivalence. One of these hypotheses emphasizes the importance of vocal learning, while the other suggests that the harmonic structure of (mammalian) vocalizations is key. Our study provides evidence suggesting that rats do indeed perceive octave equivalence. As of now, there is no evidence that rats are vocal learners, with no study observing that rats learn their ultrasonic calls (either by imitation or by active teaching), nor that rats can learn to produce the vocalizations of other animals, nor that rats can learn novel vocalizations (e.g. [19,26,38]). While we cannot exclude the possibility that future research may reveal heretofore unknown vocal learning abilities in rats, at this point, our results support the importance of vocal harmonic structure over vocal learning abilities.

More detailed questions of phylogeny remain open. From the results found here, rats emerge as the third mammalian species demonstrated to perceive octave equivalence besides bottle-nosed dolphins [14] and rhesus macaques [13]. Because none of these species are closely related, our results further support the idea that octave equivalence may be common in mammalian species, perhaps dating back to the ancestral roots of this clade. However, there is a complication here. In a previous head-turning study with common marmosets (*Callithrix jacchus*), members of our group found no evidence of octave equivalence [39]. The study used a procedure that was adapted from a study with human infants [31] using a habituation/dishabituation paradigm similar to the one used in the study presented here. Both the current study and the study with marmosets familiarized animals to tones in one octave, and then assessed attention to tones transposed by an octave versus other non-octave transpositions. Like rats, common marmosets are a mammalian species which produce harmonic vocal output and are not capable of vocal learning in the classical sense [40] of learning new sounds (despite marmoset vocal development benefiting from parental feedback [41]. Future studies should re-address the question of whether common marmosets perceive octave equivalence, perhaps by implementing the paradigm used here. Additionally, further species must be tested for octave equivalence to strengthen the idea that octave equivalence is indeed a common trait in mammals (because to our knowledge, only the four mentioned mammalian species have been tested so far). In this endeavour, the paradigm used in this study will be a helpful tool. If—after such research has been conducted—marmosets still do not show evidence of octave equivalence, but many or most other tested mammals at that point do, we will benefit from asking why. If this trait is indeed common across mammalian clades, why would it have been lost in one particular species? This would give us insight into the evolution of octave equivalence from a different perspective.

Another way to gain such insight would be by further studying birds. Two avian species (black-capped chickadees [15] and budgerigars [16]) showed no evidence of octave equivalence in studies that were using a design based on Hoeschele *et al*. [2]—just as the study at hand does. Octave equivalence has been suggested to be a by-product of harmonic fusion. This term describes the phenomenon that, despite several discrete pitches being present in harmonic sounds (the fundamental frequency and its overtones), they are perceived to be one unified sound. When multiple sounds share most or all of their harmonic information, they may thus also be perceived as one unified sound [42]. If this is the case, then under what circumstances does harmonic information become important? Perhaps for songbirds, because they often have noisy vocalizations (e.g. [43]) and/or vocalizations with more than one fundamental frequency (because of being able to independently control the two sides of their syrinx [44]), harmonic fusion may be less salient a feature. In other words, different modes of perception may arise due to differences in vocal production between mammals and avian species particularly in terms of harmonicity [18]. But the story is not so simple. We do know that some birds actively use intervals found in the harmonic series in their vocalizations [45,46] and we know at least one case where a songbird used octave equivalence during vocal learning: a house finch tutored by a canary imitated the octave information present in the canary’s vocalizations [47]. In which cases is there evolutionary benefit in attending to octave information and is octave equivalence found in a common ancestor to both mammals and birds? More research is needed to answer these questions.

For now, our results showing that rats perceive octave equivalence imply that octave equivalence may be a more basal trait which has later been co-opted in humans for both musical behaviour as well as for language learning. As described above, human children use octave equivalence to produce accurate imitations of words presented at a pitch that is too low for them to reproduce [7–9]. It is unclear whether this phenomenon is cross-cultural. Adult native Amazonian Tsimane’ showed no octave equivalence in a singing task [6], so it would be interesting whether the phenomenon occurs in their language learning. Demany & Armand [31] found octave equivalence in infants, which suggests it is likely to be found cross-culturally. As described above, cross-species studies allow for control of confounds of culture and biology in a way that is simply not possible with humans. In humans, octave equivalence has been suggested to be influenced by musical training or exposure to music, but also simply by harmonic sounds such as the human voice (e.g. [48]). Yet, it is impossible to test such notions with humans. With rats as a model species, developmental studies appear possible and could finally answer questions regarding under what circumstances octave equivalence does and does not arise in ontogeny.

## 5. Conclusion

In this study, we aimed to test concurring hypotheses on the evolution of octave equivalence highlighting either vocal learning or the harmonic structure (particularly) of mammalian vocalizations. We found strong evidence that rats perceive octave equivalence in a parallel paradigm to earlier studies with human participants. Because rats are not vocal learners, this emphasizes the importance of harmonic vocalizations for octave equivalence perception over vocal learning and points towards the importance of aspects of the perceptual and vocal production system that are shared in mammals due to phylogeny. Future cross-species studies on octave equivalence stand to fortify this idea with further evidence.

## Data Availability

Raw and processed data are available at [35].
